# Evaluation of the INTERPRET decision-support system: can it improve the diagnostic value of magnetic resonance spectroscopy of the brain?

**DOI:** 10.1007/s00234-018-2129-7

**Published:** 2018-11-15

**Authors:** J. Hellström, R. Romanos Zapata, S. Libard, J. Wikström, F. Ortiz-Nieto, I. Alafuzoff, R. Raininko

**Affiliations:** 1 Department of Radiology, Uppsala University, Uppsala, Sweden; 2 Department of Immunology, Genetics and Pathology, Uppsala University, Uppsala, Sweden; 3 Department of Pathology, Uppsala University Hospital, Uppsala, Sweden

**Keywords:** Brain, Magnetic resonance imaging, Magnetic resonance spectroscopy, Decision-support system, Computer-aided diagnosis

## Abstract

**Purpose:**

We evaluated in a clinical setting the INTERPRET decision-support system (DSS), a software generated to aid in MRS analysis to achieve a specific diagnosis for brain lesions.

**Methods:**

The material consisted of 100 examinations of focal intracranial lesions with confirmed diagnoses. MRS was obtained at 1.5 T using TE 20–30 ms. Data were processed with the LCModel for conventional analysis. The INTERPRET DSS 3.1. was used to obtain specific diagnoses. MRI and MRS were reviewed by one interpreter. DSS analysis was made by another interpreter, in 80 cases by two interpreters. The diagnoses were compared with the definitive diagnoses. For comparisons between DSS, conventional MRS analysis, and MRI, the diagnoses were categorised: high-grade tumour, low-grade tumour, non-neoplastic lesion.

**Results:**

Interobserver agreement in choosing the diagnosis from the INTERPRET database was 75%. The diagnosis was correct in 38/100 cases, incorrect in 57 cases. No good match was found in 5/100 cases. The diagnostic category was correct with DSS/conventional MRS/MRI in 67/58/52 cases, indeterminate in 5/8/20 cases, incorrect in 28/34/28 cases. Results with DSS were not significantly better than with conventional MRS analysis. All definitive diagnoses did not exist in the INTERPRET database. In the 61 adult patients with the diagnosis included in the database, DSS/conventional MRS/MRI yielded a correct diagnosis category in 48/32/29 cases (DSS vs conventional MRS: *p* = 0.002, DSS vs MRI: *p* = 0.0004).

**Conclusion:**

Use of the INTERPRET DSS did not improve MRS categorisation of the lesions in the unselected clinical cases. In adult patients with lesions existing in the INTERPRET database, DSS improved the results, which indicates the potential of this software with an extended database.

**Electronic supplementary material:**

The online version of this article (10.1007/s00234-018-2129-7) contains supplementary material, which is available to authorized users.

## Introduction

A noninvasive method to specifically diagnose brain lesions would be extremely useful. In order to improve the specificity of magnetic resonance spectroscopy (MRS), a multicentre project, funded by the European Commission, the International Network for Pattern Recognition of Tumours using Magnetic Resonance (INTERPRET), was conducted. Spectra of brain tumours from many European centres were collected into a database. With the ability to match a spectrum from a patient to those of the other patients in the database, the need for histological diagnosis could diminish. The evaluation of spectra is made with graphical software, the INTERPRET decision-support system (DSS), which now also contains some non-tumourous spectra.

The first version of the software was shown to improve the diagnosis for the most common intracranial tumours when used for MRS analysis compared to MRI alone [1]. A prospective study of brain tumour cases showed that the INTERPRET DSS outperformed three other systems for spectra classification. That study also proved that the added information from spectral data could improve the radiologist’s diagnosis compared to a diagnosis made with MRI alone [2].

In the second version, the INTERPRET DSS 2.0, an option to use both short and long TEs, was introduced. This, however, did not improve the diagnostic accuracy, but the study showed that neuroradiologists inexperienced in use of MR spectroscopy could use the DSS with outcomes similar to those of experienced spectroscopists [3]. Another study demonstrated that the use of a combination of short and long TEs with the INTERPRET DSS could help in the differential diagnosis between glioblastoma multiforme and metastasis [4]. In another study, use of the INTERPRET DSS 2.0 did not improve the classification of brain tumours as high-grade or low-grade, compared to analysis of spectra by spectroscopists [5].

**Fig. 1:**
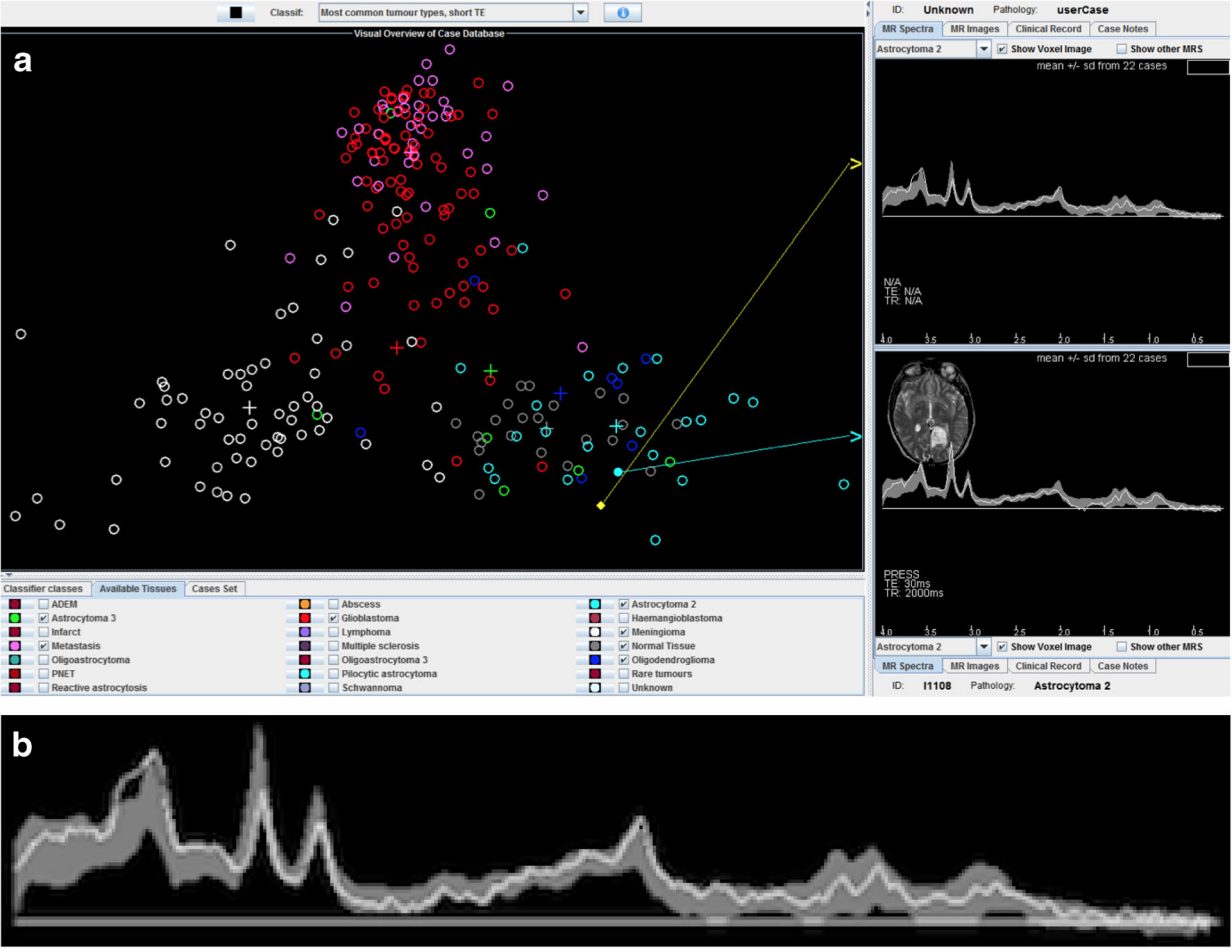
Graphical user interface in the INTERPRET DSS 3.1 (**a**). The circles in the main frame represent cases in the database. The spectrum at the lower right is a spectrum from the database of a patient with astrocytoma grade II. The spectrum at the upper right is a study patient’s spectrum (magnified in (**b**)). Grey background showing mean ± SD of 22 spectra of astrocytoma grade II

In the version 3.0 of the INTERPRET DSS, the software has the objective of being able to analyse new data from any MR manufacturer, and this can be done by a person with even minimal knowledge of spectroscopy [6]. An example of the graphical user interface from the software is seen in Fig. 1.

The objective of our study was to evaluate the diagnostic utility of the INTERPRET DSS 3.1 (http://gabrmn.uab.es/dss) in a clinical setting and compare the results to those achieved on MRI and MRS without using a DSS, which will be called conventional MRS analysis in this article.

## Materials and methods

### Materials

The clinical archive of the radiology department (i.e. examinations for research purposes only were not included) was searched for MR spectroscopy of the brain performed from January 2004 to April 2014. In clinical cases, MRS was performed if the diagnosis was unknown or uncertain and information from other examinations including MRI was not sufficient for treatment planning. The study plan was approved by the regional ethics committee. To read and use patient data from the hospital archive for research purposes, written informed consent was needed from all living patients and parents of underage patients. Only patients above 13 years of age with focal lesions, a high-quality single voxel spectrum (SVS) with available raw data and a definitive diagnosis in their medical charts valid at the time of examination were included.

This left a total of 100 examinations in 97 patients to be included in the study. Three patients had undergone two examinations because of new lesions or of altered appearances in known lesions. The clinical indications for MRS are shown in Table 1.

**Table 1:** Indications for MRS

Clinical question	No. of cases
Tumour grading	37
Neoplastic vs non-neoplastic lesion (infection, inflammation, ischaemia, etc.)	20
Recurrent tumour vs reaction to irradiation/chemotherapy	20
Lesion of unknown aetiology on MRI	13
Metastatic disease vs primary tumour	7
Type of a non-neoplastic lesion	1
Metabolic disease	1
Biopsy planning (hot spot)	1
Total	100

### Radiological evaluation

An experienced neuroradiologist, blinded to the definitive diagnoses, reviewed both the MR images and MRS curves and other MRS analyses. Evaluation of the examinations was performed in the same way as is done in the normal clinical routine at our hospital, i.e. all radiological examinations obtained before and at the time of MRS were available. Clinical information to the radiologist by the referring clinician was used if available. MR images were evaluated first without MRS data. In the second evaluation, MRS data were added.

### Radiological technique

The MR imaging technique varied over the years and according to the indication for the examination. Diffusion-weighted images were often included, and perfusion MRI has been performed routinely in cases of suspected tumour for the last 5 years. T2*-weighted gradient echo sequences or SWI sequences were used in cases with suspected blood products or calcium.

Proton MRS technique was individualised according to MRS indication and case-specific questions. The patient’s clinical state, co-operation and total MR examination time were also taken in account. We tried to avoid anaesthesia. In 2004–2005, a Philips Intera (Philips Healthcare, Best, the Netherlands) imager was mainly used. Since 2005, the spectra were obtained with a Siemens Avanto (Siemens Medical Systems, Erlangen, Germany) imager. Both imagers operated at 1.5 T using PRESS sequences for single voxel spectroscopy. When the spectra were obtained with 1.5 T Philips Intera, a repetition time/echo time (TR/TE) of 6000/20–22 ms was used, which was 5000/30 ms when the Siemens Avanto was used. Additional chemical shift imaging was performed in 74 cases using TR/TE 1500–2500 ms/135–144 ms. In a few of the rest of the cases, an additional SVS with the TR/TE 1500/135–144 ms was obtained to demonstrate if there was an inverted peak in lactate position.

At SVS, the voxel was placed in the lesion seen on MRI. The size and form of the voxel was individually adjusted in order to select a representative sample. If there was contrast enhancement, the voxel was placed in that area. The voxel was placed in solid tissue, avoiding necrotic areas and cerebrospinal fluid. Fat contamination and areas with susceptibility disturbances were avoided. To detect the areas with susceptibility disturbances, T2*-weighted gradient echo or SWI sequences were used before MRS in patients who had undergone operations or had a lesion close to the skull base. Sixteen unsuppressed water reference acquisitions were obtained for quantifications. An unsuppressed water signal was used as an internal reference when metabolite concentrations were estimated.

For conventional MRS analysis, the data were processed using the LCModel. Routinely, we restricted the model to the range 0.2–4.0 ppm. The spectra were corrected for eddy currents. All spectra were manually assessed to exclude obvious non-randomness in the residuals or erroneous assignment of metabolites. Post-processing was made by an MR physicist. Examinations of low spectral quality, e.g. those with signal-to-noise ratio (SNR) < 5, were excluded from the material. The metabolites included in the diagnostic analyses had a Cramer-Rao lower bound (CRLB) ≤ 20 with exception of lactate. The presence of lactate was considered to be real if there was a clear inverted doublet peak in the spectrum with a semilong TE even if the CRLB was > 20. Millimolar metabolite concentrations (mM, millimoles/litre substance) were measured using tissue water as a reference. Ratios were routinely calculated using total creatine (Cr) as a reference but also other ratios, like N-acetylaspartate/choline (NAA/Cho), were used as diagnostic tools. We mainly used metabolite ratios since the absolute concentrations are calculated assuming that water concentration of the brain tissue is constant, which cannot always be expected. Our own control material of healthy volunteers examined with the same SVS technique and analysis method was utilised in evaluations.

On CSI, the examined area covered the pathological area but also normal or suspected normal contralateral tissue. The section thickness was 15 mm and the nominal voxel sizes 10 × 10 × 15 mm^3^. Saturation bands were placed for suppression of osseous, fatty and air-containing structures in surroundings. All data post-processing was performed by an MR physicist with softwares provided by the MR imager manufacturers and spectra at 1.1–3.5 ppm were analysed. All voxels were analysed but those with low spectral quality were not used in diagnostic purposes. In good quality spectra, at least the upper halves of the choline peaks could be separated from the creatine peaks. Metabolite ratios to Cr and Cho were calculated routinely and used in diagnostic analyses. Colour maps overlaid on the anatomical images were made routinely for metabolites Cho, Cr, NAA and lactate and for metabolite ratios using Cr and Cho as references.

MRS interpretations were made with guidance of the criteria in literature. Metabolite ratios to differentiate benign from malignant tumours on SVS have been reported by Bulakbasi et al. [7]. In a meta-analysis by Usinskiene et al. [8], a cut-off value of 1.56 for the Cho/Cr ratio at short TE obtained the highest area under curve value. A Cho/NAA threshold of 1.66 has been suggested on CSI [9]. Presence of increased lipids and lactate and a reduced NAA peak indicate a grade IV tumour [10]. For the differentiation between tumour recurrence and radiation reaction, Cho/Cr or Cho/NAA ratios of 1.7–1.9 have shown to predict a tumour, mostly on CSI [11] while a Cho/Cr threshold of 1.1 and a Cho/NAA threshold of 1.17 have been suggested on SVS with TR/TE 6000/30 [12].

### INTERPRET DSS analysis

An MRS physicist with experience with the INTERPRET DSS trained both a radiology resident with limited experience and a radiologist to use the software. The spectroscopic data from SVS with a short TE were processed through jMRUI 4.0, with removal of the water signal, before being imported into the INTERPRET DSS 3.1 (Fig. 1). All cases were examined by the radiology resident. Eighty of the 100 examinations were also evaluated by the radiologist. Both the importation process of raw spectroscopic data through jMRUI into the DSS 3.1 and the evaluations were made independently by the two analysers. The radiological referrals including the clinical indication for the examination were available but no other patient data. The study patient’s spectrum was first checked against the individual spectra of neighbouring cases in the visual overview of the cases in the database. Then, the patient’s spectrum was checked against all available tissue spectra via the overlay of spectra ± standard deviation. For the overlay that best included the patient’s spectrum, that diagnosis was chosen. In ambiguous cases, a second check against individual spectra that was close in the visual overlay could be performed. Only one diagnosis was chosen as the most likely one.

Differential diagnosis between a recurrent tumour and reaction to treatment, including irradiation and/or chemotherapy, was a common indication for MRS. No cases of such reactive changes are included in the INTERPRET database. Consequently, we used an indirect diagnostic method. If tumour tissue was found, the diagnosis was a recurrent tumour. If no neoplasm was suggested in the DSS analysis, we interpreted that we had excluded the tumour recurrence.

### Confirmation of diagnosis

The diagnoses obtained by the INTERPRET DSS were compared with the definitive diagnoses. Confirmation of the definitive diagnosis was neuropathological in 61 lesions, from long-term follow-up in 36 cases, and biochemical or genetic in three cases.

The median time from radiological examination to lesion sampling for neuropathological analyses was 17 days (range 1–222 days, upper quartile 39 days). The possible effect of the time lapse from the radiological examinations to sampling was considered for each case before inclusion in the study. The material was processed at the department of pathology following a standard routine including the use of histochemical (haematoxylin-eosin (HE)) and immunohistochemical techniques. All original HE-stained slides were reassessed by two neuropathologists. The grading of tumours followed the World Health Organisation (WHO) Classification of Tumours of the Central Nervous System [13]. Briefly, all cases with at least one mitotic figure in HE sections were graded as high-grade tumours. The diagnosis was considered as a neuropathologically verified astrocytoma grade II for one patient with a previous neuropathological diagnosis of astrocytoma grade I–II with recurrent tumour and a clinical course not consistent with a high-grade tumour. The diagnosis was also considered as a neuropathologically verified astrocytoma grade III for two patients where the neuropathological diagnosis was grade II–III and the clinical course matched grade III. For two patients with a histologically confirmed glioblastoma at primary surgery, followed by a fast-growing lesion in the same location and a rapid deterioration of clinical condition, the diagnosis was considered neuropathologically verified.

In 36 cases, a diagnosis was made during a follow-up of at least 6 months, during which time imaging findings, other examinations, and the patients’ clinical condition were evaluated.

### Comparison of diagnoses made with the DSS, conventional MRS and MRI

Results with the INTERPRET DSS were compared with results yielded by MRS with conventional analysis combined with MRI and by MRI alone. For statistical comparisons, the diagnoses were divided into three clinically relevant categories: primary high-grade CNS tumours (WHO Gr III–IV) and metastases, low-grade tumours (WHO Gr I–II), and non-neoplastic lesions.

The DSS, conventional MRS, and MRI diagnoses were classified as follows:

- Correct: The diagnosis was categorised correctly.
- Indeterminate: The diagnosis was categorised correctly, but also an incorrect category was given as an alternative in conventional analysis. In the DSS analysis, this classification was used when there was no good match in the database.
- Incorrect: The diagnosis was categorised incorrectly.

### Statistical analysis

The results for the different diagnostic categories were compared using a *χ*^2^ test.

The interobserver agreement for the DSS was evaluated using kappa statistics.

## Results

### Interobserver agreement

The same diagnosis was chosen in 60/80 (75%) of the cases when the two readers independently analysed the cases in the INTERPRET DSS 3.1. This gives a kappa value of 0.71, representing substantial agreement. The most common discrepancy in answers was between metastasis and glioblastoma, which occurred in seven cases. The chosen diagnoses fell in the same category in 72/80 cases (90%, Cohen kappa value of 0.84 (almost perfect agreement) [14]).

### Radiological outcome

Of the 100 cases, 56 were high-grade tumours, 20 low-grade tumours, and 24 were non-neoplastic lesions (Tables 2, 3 and 4). Compared with the definitive diagnoses, the diagnoses from the INTERPRET DSS were correct in 38/100 cases (38%), incorrect in 57/100 (57%), and indeterminate (no good match found) in five cases (5%). In seven cases, the definitive diagnosis was low-grade glioma without specific tumour typing due to unsuccessful or missing biopsy, and in all, the INTERPRET DSS yielded a specific diagnosis of some low-grade glioma. Thus, there were no diagnostic discrepancies, but it is impossible to know whether the diagnoses were correct. If those seven cases were excluded, the DSS would have yielded a correct diagnosis in 31/93 (33%) cases, and the number of incorrect diagnoses would have been 57/93 (61%).

**Table 2:** Outcome of diagnoses made with the INTERPRET DSS 3.1 for the high-grade tumours

Definitive diagnosis	*N*	DSS dg				Confirmation of diagnosis
		Correct *N*	Incorrect	*N*	No good match *N*	
Glioblastoma	28	13		13	2	Neuropathological 25
						Clinical follow-up 3^b^
			Astrocytoma Gr III	4		
			Metastasis	3		
			Astrocytoma Gr II	2		
			PNET	1		
			Schwannoma	1		
			Oligoastrocytoma	1		
			Lymphoma	1		
Astrocytoma Gr III	16	4		11	1	Neuropathological 13
						Clinical follow-up 3^c^
			Astrocytoma Gr II	3		
			Oligodendroglioma	2		
			Glioblastoma	2		
			Lymphoma	2		
			Oligoastrocytoma	1		
			Normal	1		
Lymphoma	4	1		3		Neuropathological 4
			Astrocytoma Gr III	1		
			Glioblastoma	1		
			Metastasis	1		
Metastasis	4	2		2		Neuropathological 4
			Lymphoma	1		
			Glioblastoma	1		
Gliomatosis cerebri Gr III^a^	2			2		Neuropathological 2
			Astrocytoma Gr II	1		
			Normal	1		
Gliosarcoma^a^	2			2		Neuropathological 2
			Metastasis	1		
			Glioblastoma	1		
Total	56	20		33	3	Neuropathological 50
						Clinical follow-up 6

The clinical follow-up was at least 6 months

^a^Diagnosis not found as diagnostic option in the INTERPRET DSS 3.1

^b^One patient had a tumour called glioblastoma in medical records from abroad, but no neuropathological reassessment was made; the second patient had a multifocal brain tumour; but no extracranial primary tumour was found and no neuropathological diagnosis was obtained; and the third patient had a previous neuropathological diagnosis of astrocytoma grade III with clinical tumour progression

^c^Three patients who had a neuropathological diagnosis of astrocytoma grade II prior to the radiological examination with tumour recurrence and a clinical course best matching astrocytoma grade III. In one of these patients, a new biopsy 8 months after the radiological examination gave a neuropathological diagnosis of glioblastoma

**Table 3:** Outcome of diagnoses made with the INTERPRET DSS 3.1 for the low-grade tumours

Definitive diagnosis	*N*	DSS dg				Confirmation of diagnosis
		Correct *N*	Incorrect	*N*	No good match *N*	
Astrocytoma Gr II	8	4		3	1	Neuropathological 8
			Oligodendroglioma	1		
			Oligoastrocytoma	1		
			Rare tumours^b^ (ependymoma)	1		
Oligodendroglioma	2	1		1		Neuropathological 2
			Astrocytoma Gr II	1		
DNET^a^	1			1		Clinical follow-up 1
			Normal	1		
Ganglioglioma^a^	1			1		Clinical follow-up 1
			PNET	1		
Meningioma	1			1		Neuropathological 1
			Glioblastoma	1		
Total for specified low-grade tumours	13	5		7	1	Neuropathological 11
						Clinical follow-up 2
Low-grade glioma, not specified^a^	7	7^c^	DSS dg			Clinical follow-up 7
			Astrocytoma Gr II *N = 6*			
			*N* Oligoastrocytoma *N = 1*			
			*N*			
Total (all low-grade tumours)	20	12		7	1	Neuropathological 11
						Clinical follow-up 9

The clinical follow-up was at least 6 months

^a^Diagnosis not found as diagnostic option in the INTERPRET DSS 3.1

^b^Rare tumour group consisting of rare benign and malignant tumours. For this case, the spectrum best matched an ependymoma in that group

^c^Specific diagnosis is missing—therefore unknown whether the specific DSS diagnoses were correct, but they were diagnosed as low-grade gliomas

**Table 4:** Outcome of diagnoses made with the INTERPRET DSS 3.1 for the non-neoplastic lesions

Definitive diagnosis	*N*	DSS dg				Confirmation of diagnosis
		Correct *N*	Incorrect	*N*	No good match *N*	
Reaction to irradiation/chemotherapy^a^ (exclusion of tumour tissue)	14	2^**c**^		12		Clinical follow-up 14
			Astrocytoma Gr II	6		
			Glioblastoma	3		
			Astrocytoma Gr III	2		
			Oligoastrocytoma	1		
Normal^b^	4	4				Clinical follow-up 4
Inflammation/de-myelinisation^a^	4			4		Neuropathological 1
						Biochemical 3
			Normal	4		
Abscess	1				1	Clinical follow-up 1
Ischemia^a^	1			1		Clinical follow-up 1
			Normal	1		
Total	24	6		17	1	Clinical follow-up 20
						Biochemical 3
						Neuropathological 1

The clinical follow-up was at least 6 months

^a^Diagnosis not found as diagnostic option in the INTERPRET DSS 3.1

^b^Final diagnosis: exclusion of a neoplasm in cases with focal lesions, which in many cases regressed spontaneously

^c^Two cases with normal spectrum as DSS diagnosis were categorised as correct because neoplasm was excluded

The definitive diagnosis was not available as a diagnostic option in the INTERPRET database in 32/100 cases. We repeated the same calculations only in cases with a definitive diagnosis existing in the database and also excluded 7 patients younger than 18 years because the validated classifiers in the database were not trained for them. The DSS yielded a correct diagnosis in 28/61 cases (46%). Among those 50 cases in which the diagnosis was confirmed by histopathology and available as an option in the DSS in adult patients, the INTERPRET DSS identified the correct diagnosis in 23 (46%).

The distribution of the diagnoses made with the DSS on a diagnosis category level can be seen in Tables 5 and 6. In all 100 cases, the diagnostic category (high-grade tumour, low-grade tumour, non-neoplastic lesion) given by the INTERPRET DSS was correct in 67 cases, indeterminate in 5, and incorrect in 28 cases. About 20%, the high-grade tumours had fallen into the category of low-grade tumours. However, good results on a category level were achieved for the high-grade tumours such as lymphoma and metastasis, but the differentiation between metastatic and primary high-grade tumours and between high-grade gliomas and lymphomas was not successful (Table 2). Low-grade gliomas, both specified and unspecified, were almost all classified in the correct category. By contrast, about one half of the non-neoplastic lesions are interpreted as tumours, half of which high-grade tumours (Table 5). Exclusion of neoplastic tissue in patients with reaction to irradiation/chemotherapy was unsuccessful; the spectrum was found to be most consistent with a neoplasm in 12/14 examinations (Table 4). When excluding diagnoses not included in the INTERPRET DSS, for the non-neoplastic lesions all cases of incorrect category placement were reduced to zero (Table 6). If the diagnosis was as an option in the INTERPRET database, the category was right in 79% (Table 6). The most common incorrect placement into a diagnosis category are high-grade tumours which are interpreted as low-grade tumours.

**Table 5:** Diagnostic outcome with use of the INTERPRET DSS on a diagnosis category level. Total material

Definitive diagnosis category		Diagnosis category using INTERPRET			
	No. of cases	Correct	Incorrect	*N*	No good match
High-grade tumour	56	40	Low-grade tumour	11	3
			Non-neoplastic lesion	2	
Low-grade tumour	20	16	High-grade tumour	2	1
			Non-neoplastic lesion	1	
Non-neoplastic lesion	24	11	High-grade tumour	5	1
			Low-grade tumour	7	
Total	100	67		28	5

**Table 6:** Cases with diagnoses not existing in INTERPRET DSS 3.1 excluded. Patients under 18 years of age also excluded

Definitive diagnosis category		Diagnosis category using INTERPRET			
	No. of cases	Correct	Incorrect	*N*	No good match
High-grade tumour	48	38	Low-grade tumour	7	3
			Non-neoplastic lesion		
Low-grade tumour	8	6	High-grade tumour	1	1
			Non-neoplastic lesion		
Non-neoplastic lesion	5	4	High-grade tumour		1
			Low-grade tumour		
Total	61 (100%)	48 (79%)		8 (13%)	5 (8%)

The outcome for the INTERPRET DSS, for conventional analysis with MRS combined with MRI, and for MRI alone as tools for placing the lesion in a diagnostic category is shown in Table 7. There was no significant difference in results when the outcomes correct versus the others were compared between the DSS and conventional MRS (*p* = 0.19). The DSS was significantly better than MRI alone (*p* = 0.03), but conventional MRS analysis was not. Excluding diagnoses not found in the INTERPRET DSS, results for the same outcomes in patients at least 18 years of age, were significantly better for the DSS compared to conventional MRS analysis and MRI (Table 8, *p* values 0.002 and 0.0004, respectively).

**Table 7:** Diagnostic outcome on a category level with use of the INTERPRET DSS, with conventional interpretation of MRS, and with MRI alone. Diagnostic categories: high-grade tumour, low-grade tumour and non-neoplastic lesion. Total material

Diagnostic outcome	INTERPRET DSS	MRI + MRS	MRI
Correct	67 (67%)^ab^	58 (58%)^a^	52 (52%)^b^
Indeterminate	5 (5%)	8 (8%)	20 (20%)
Incorrect	28 (28%)	34 (34%)	28 (28%)
Total	100 (100%)	100 (100%)	100 (100%)

^a^*p* value = 0.19 when the outcomes correct vs others are compared for the DSS and MRI + MRS

^b^*p* value = 0.03 when the outcomes correct vs others are compared for the DSS and MRI

**Table 8:** Cases with diagnoses not existing in INTERPRET DSS 3.1 excluded. Patients under 18 years of age also excluded

Diagnostic outcome	INTERPRET DSS	MRI + MRS	MRI
Correct	48 (79%)^ab^	32 (53%)^a^	29 (48%)^b^
Indeterminate	5 (8%)	5 (8%)	14 (23%)
Incorrect	8 (13%)	24 (39%)	18 (29%)
Total	61 (100%)	61 (100%)	61 (100%)

^a^*p* value = 0.002 when the outcomes correct vs others are compared for the DSS and MRI + MRS

^b^*p* value = 0.0004 when the outcomes correct vs others are compared for the DSS and MRI

Additional data are given in Online Resource 1 for the diagnoses which are included in the validated classifiers in the INTERPRET database [1]. In that more selected material, a correct category was yielded by the DSS in 32/38 cases, by conventional MRS in 21/38 cases and by MRI in 18/38 cases.

## Discussion

To our knowledge, this material is the largest group of unselected clinical patients in which the diagnostic outcome using the INTERPRET DSS 3.1 compared to conventional MRS and MRI analysis has been studied until now. In our material of focal lesions, a correct specific diagnosis was made with the use of the INTERPRET DSS 3.1 in about one-third of the lesions. The diagnosis fell into the right category (high-grade tumours, low-grade tumours or non-neoplastic lesions) in two-thirds of the lesions.

The ability to provide specific diagnoses varied in different tumours. The discrimination between glioblastoma and metastasis can be particularly difficult. This is reflected in the interobserver agreement results in this study and has also been shown in attempts to separate these spectra in the literature [15, 16]. For the diagnosis anaplastic astrocytoma, only one-quarter of the cases were given the correct specific diagnosis. The reason could be that this tumour can be difficult to differentiate from other tumours with regard to cellular architecture and molecular patterns, also for the pathologist [17, 18]. A recent study using cases from the INTERPRET database showed that even for the binary classification of anaplastic astrocytoma compared to other tumour grades or healthy tissue, an area under curve > 0.9 in receiver operating characteristics operating analysis was infrequently achieved at 1.5 T with a short TE [19]. In an earlier study on the other hand, the use of INTERPRET was better than MRI for characterisation of anaplastic astrocytoma [2]. Further development of the classifiers in the INTERPRET database could improve classification of glial tumour grades [20].

In this unselected clinical case material, about one-third of the definitive diagnoses did not exist in the INTERPRET database, which influenced the possibility to find the correct diagnosis. The material in the database consists mainly of tumours, but even some tumours, for example, gliosarcoma, gliomatosis cerebri, and ganglioglioma, were missing or grouped together as rare tumours. To test the potential effectiveness of the method, we also did analyses of only cases where the definitive diagnoses were in the database. Not more than 46% of those cases were given the correct specific diagnosis, and the diagnostic category (high-grade tumour, low-grade tumour, non-neoplastic lesion) was correct in 79%. In the whole material, the category was correct in 67% of the lesions. The size of the INTERPRET database is thus a limiting factor. However, even in cases where the specific diagnosis is not an option in the INTERPRET database, exclusion of a certain diagnosis can be valuable, but, by contrast, an incorrect diagnosis may lead to serious consequences. For example, in 12/14 (86%) of reactions to irradiation/chemotherapy, the interpretation was a neoplastic spectrum, a misclassification which can influence patient care.

In our study, the use of the INTERPRET DSS 3.1 did not improve results in choosing the right diagnostic category (high-grade tumour, low-grade tumour, non-neoplastic lesion) for the total material compared to conventional MRS but did so compared to MRI (67% vs. 58% and 52%, respectively). Our results are in line with those of previous studies [1, 2] in which use of the INTERPRET DSS improved the diagnoses compared to MRI. When excluding diagnoses not found in the database, our outcome for the DSS was significantly better than for conventional MRS, marking the possible potential with a larger database of more diagnoses.

With the use of the DSS, the numerical value of indeterminate diagnoses was reduced. This could be due to the fact that, according to the study design, only one diagnosis was selected from the INTERPRET database. This could, however, also have resulted in increasing the number of incorrect diagnoses.

Interpreters can learn to use the INTERPRET DSS software with substantial agreement in diagnoses in a short period of time. Our results are in agreement with the aim of the software to be useful even for users with minimal knowledge of MR spectra [6]. Differences in interpretation can arise when interpreters differ with regard to which peak is the most important to fit within the example spectra in the database. In the clinical routine at our hospital, it is an MR physicist who independently makes an interpretation of the spectrum in the INTERPRET DSS and presents it to the radiologist, who combines it with his/her own findings from MRI and conventional MRS analysis.

We used imagers operating at 1.5 T, and the INTERPRET classifiers were based on 1.5 T datasets. A study by Garcia-Fuster et al. [21] suggests that it is possible to also use data from spectra acquisitions at 3 T. This has been tested with good results in a few patients, also by Julia-Sape et al. [20]. The use of 3 T field strength allows for better metabolite separation [22].

MRS is often used in cases when MRI is ambiguous. This may be an explanation for the rather low accuracy for all radiological methods in this study because cases that were easy to diagnose were not included. Interpreting MRI and MRS at the same time can affect the interpretation of both examinations. In cases where the diagnosis was made on the basis of clinical follow-up, the interpretation of the MR examinations may have had an influence on the clinical diagnosis.

We used a longer TR than in the cases included in the database (1600–2000 ms) [1]. It is unclear if that would affect the classifier performance. In a study of healthy study subjects by Knight-Scott et al. [23], no difference in relative metabolite concentrations was seen for longer TRs (TR > 2500 ms). The number of cases in the INTERPRET database was a limiting factor. Another limiting factor was that not all non-neoplastic lesions or low-grade tumours had a histological diagnosis. This results in non-specific diagnoses and can affect whether the diagnosis exists in the INTERPRET database or not. Both tumours and post-therapy areas may be heterogeneous. The sites of the MRS voxels and samples taken for neuropathological examination may be suboptimal and not exactly the same. For 75% of the cases in our study with a neuropathological diagnosis, the tissue sample was obtained within 40 days of the radiological examination. Some samples were obtained after several months after radiologic examination, which could be acceptable in the case of slowly altering/benign disease as well as in cases where the primary radiological diagnosis could be classified unambiguously.

The possible future for the INTERPRET project is outlined in a review article from 2015 [20], which includes inclusion of children’s spectra, spectra at higher field strengths, even more tumour types, and also non-neoplastic diseases. To be seamlessly integrated into the everyday workflow would be advantageous for the DSS. Another possible development is to automatically classify the spectrum without user input to make the diagnoses reproducible. Cases from the INTERPRET database are also included in the larger eTumour database [24]. Measures to make sure that cases in the INTERPRET database are validated have been taken, but 10% of cases that had passed quality control were discarded when evaluated by expert spectroscopists [25].

## Conclusion

The INTERPRET DSS software can be used with substantial agreement between interpreters after a short training period. In an unselected clinical material, a correct specific diagnosis was obtained in less than 40% of the focal lesions and a right category (high-grade tumours, low-grade tumours, and non-neoplastic lesions) in 67%. The INTERPRET DSS did not improve the categorising of the lesions significantly compared to conventional analysis of MRS, but did so compared to MRI alone. Excluding diagnoses not found in the INTERPRET DSS, results for the same outcomes were significantly better for the DSS compared to conventional MRS analysis and MRI. The right category was obtained in 79%. The size of the INTERPRET database is a limiting factor because only two-thirds of the definitive diagnoses were available in the database. Further improvement in the accuracy and reproducibility of this software may be anticipated, with the addition of more diagnoses in the database and full automatisation of the evaluation.

## Electronic supplementary material

ESM 1 (PDF 105 kb)

## Abbreviations

Cho: Choline
Cr: Creatine
CSI: Chemical shift imaging
DSS: Decision-support system
INTERPRET: International Network for Pattern Recognition of Tumours Using Magnetic Resonance
MRS: Magnetic resonance spectroscopy
NAA: N-acetylaspartate
PRESS: Point-resolved spectroscopy
SVS: Single voxel spectroscopy
TR: Time to repetition
TE: Time to echo
WHO: World Health Organisation
