# Supplementary material for: Evaluation of the INTERPRET decision-support system: can it improve the diagnostic value of magnetic resonance spectroscopy of the brain?
Source: Neuroradiology. 2018 Nov 15;61(1):43–53. doi: 10.1007/s00234-018-2129-7 (PMC6336758; doi:10.1007/s00234-018-2129-7)
Supplement: Supplementary file 1 — (PDF 105 kb) [file 234_2018_2129_MOESM1_ESM.pdf]

Electronic Supplementary Material

Evaluation of the INTERPRET Decision Support System. Can it improve the diagnostic value of magnetic resonance spectroscopy of the brain?

Neuroradiology

J. Hellström[1*], R. Romanos Zapata[1], S. Libard[2,3], J. Wikström[1], F. Ortiz-Nieto[1], I. Alafuzoff[2,3], R. Raininko[1]

[1]Department of Radiology, Uppsala University, Uppsala, Sweden
[2]Department of Immunology, Genetics and Pathology, Uppsala University, Uppsala, Sweden
[3]Department of Pathology, Uppsala University Hospital, Uppsala, Sweden

Corresponding author E-mail: jussi.hellstrom@radiol.uu.se

Online Resource 1 Table. Diagnostic outcome with use of the INTERPRET DSS, with conventional interpretation of MRS, and with MRI alone for the diagnoses included in the validated tumour classifiers in the DSS (http://gabrmn.uab.es/dss) [1] for patients at least 18 years of age. Diagnostic categories: high-grade tumour, low-grade tumour, and non-neoplastic lesion.

**Validated INTERPRET classifier**	**Specific diagnosis**	**N**	**DSS**				**MRI + MRS**				**MRI**		
			IC	ID	CO		IC	ID	CO		IC	ID	CO
Low grade meningioma	Meningioma	1	1	0	0		0	1	0		0	0	1
Low grade glial tumour	Astrocytoma II	5	0	1	4		2	1	2		4	0	1
	Oligodenglioma	2	0	0	2		1	0	1		0	1	1
	Oligoastrocytoma	0	0	0	0		0	0	0		0	0	0
Aggressive tumor	Glioblastoma	26	2	2	22		10	1	15		7	7	12
	Metastasis	4	0	0	4		1	0	3		1	0	3
**Total**	%	**38**<br>**100**	**3**<br>**8**	**3**<br>**8**	**32**<br>**84**		**14**<br>**37**	**3**<br>**8**	**21**<br>**55**		**12**<br>**32**	**8**<br>**21**	**18**<br>**47**

Abbreviations
IC Incorrect category

ID Indeterminate category (No good match in the DSS/Correct but also incorrect category given in MRI or MRI+MRS)

CO Correct category

1. Tate AR, Underwood J, Acosta DM, et al. (2006) Development of a decision support

system for diagnosis and grading of brain tumours using in vivo magnetic resonance single voxel spectra. NMR Biomed 19:411–34. doi: 10.1002/nbm.1016
